# Understanding the community management of long-term physical and mental health conditions in Bolivia, Colombia and Guatemala: a situational analysis

**DOI:** 10.1136/bmjgh-2025-020466

**Published:** 2026-03-09

**Authors:** Juan Camilo Marin-Urrego, Adriana Buitrago-Lopez, Carlos Gomez-Restrepo, Edgar Lopez Alvarez, Ronald Fernando Tapia Pijuan, Lucia Elena Alvarado-Arnez, Estela Tango-Camargo, Yazmin Cadena-Camargo, David Niño-Torres, Nelcy Rodriguez-Malagon, Isabela Osorio Jaramillo, Shirley Nicole Andrade Azcui, Patricia Cabaleiro, James Yhon Robles Pinto, Luis Felipe Osinaga Robles, Luis Padilla-Vassaux, Carmen Maria Sanchez-Nochez, Candelaria Letona, Victoria Jane Bird

**Affiliations:** 1 Department of Clinical Epidemiology and Bioestatistics, Pontificia Universidad Javeriana, Bogotá, Colombia; 2 Hospital Universitario San Ignacio, Bogotá, Colombia; 3 Department of Psychiatry and Mental Health, Pontificia Universidad Javeriana, Bogotá, Colombia; 4 Facultad de Ciencias de la Salud, Universidad Rafael Landivar, Guatemala City, Guatemala; 5 Facultad de Ciencias de la Salud Santa Cruz, Universidad Privada Franz Tamayo, Santa Cruz de la Sierra, Bolivia; 6 Department of Preventive and Social Medicine, Pontificia Universidad Javeriana, Bogotá, Colombia; 7 Institute of Public Health and Wellbeing, University of Essex, Colchester, UK; 8 Wolfson Institute of Population Health, Queen Mary University of London, London, UK

**Keywords:** Mental Health & Psychiatry, Health Services Accessibility, Delivery of Health Care, Cardiovascular disease, Community-based survey

## Abstract

**Introduction:**

Community-based healthcare approaches can improve outcomes and reduce costs for long-term physical and mental health conditions. To design, evaluate and implement such interventions, it is essential to explore the existing resources of community and healthcare institutions, understand stakeholder perspectives and identify potential barriers and facilitators to community-based care for non-communicable diseases. Our aim was to conduct a situation analysis to better understand and contextualise community-based care for long-term physical and mental health conditions in Bolivia, Colombia and Guatemala.

**Methods:**

A multimethod approach was used, incorporating three data sources: (1) sociodemographic and morbidity indicators from selected regions and healthcare centres; (1) quantitative surveys completed by health centre management staff and (2) semistructured interviews with healthcare workers, patients, caregivers and community leaders. These tools helped assess the capacity of health centres, as well as barriers and facilitators for community-based care. Data were analysed using descriptive statistics and thematic framework analysis.

**Results:**

25 health centres across the three countries were included: 12 were of low complexity, 21 in urban areas and 20 used electronic medical records. Daily seen patients ranged from 1 to 270. Most of the centres had general practitioners and nursing staff, with 72% having psychologists, 24% psychiatrists and 50% specialists in cardiovascular or metabolic conditions. Barriers to community-based care included duration and frequency of appointments, a shortage of both administrative and clinical staff, a lack of continuity in treatment, long distances for patients to travel, inadequate facilities and mental health stigma.

**Conclusion:**

Community interventions aim to manage long-term physical and mental health conditions; however, identified barriers may limit their implementation within the existing healthcare infrastructure and should be addressed when introducing new approaches.

**WHAT IS ALREADY KNOWN ON THIS TOPIC**

- Non-communicable diseases (NCDs), including mental health conditions, pose a major challenge for low- and middle-income countries, where most premature deaths from NCDs occur. The prevention and management of these long-term conditions require community-based efforts.

**WHAT THIS STUDY ADDS**

- This study provides insight into barriers and facilitators of community-based care in health centres in three Latin American countries and proposes a methodological approach for conducting a situation analysis, focusing on implementation, specifically of low-cost community-based interventions.

**HOW THIS STUDY MIGHT AFFECT RESEARCH, PRACTICE OR POLICY**

- This work has implications for the future implementation of effective and low-cost community-based interventions aimed at improving the health outcomes and quality of life of people living with long-term NCDs, including those related to mental health conditions in Latin America.

## Introduction

 Non-communicable diseases (NCDs), including both physical and mental health conditions, represent a significant challenge to healthcare systems, individuals, families and communities in low- and middle-income countries (LMICs).^1 2^ Each year, 17 million people under the age of 70 years die from NCDs, with 86% of these premature deaths occurring in LMICs.^1^ These conditions are largely preventable, with up to 80% of associated deaths preventable if primary risk factors, such as smoking, unhealthy diet, physical inactivity and harmful alcohol consumption, are effectively addressed.^2^ Furthermore, mental health issues have increased following the COVID-19 global pandemic, including limited access to mental health specialists for individuals with severe mental illnesses.^3^

The burden caused by NCDs may be reduced through resource-orientated low-cost community-based interventions that operate at different levels within the healthcare system, from primary care to highly specialised care. These interventions aim to support patients in drawing from existing resources within themselves and their relationships with their families, wider social networks and communities to reduce distress and improve quality of life.^4^,^6^ The potential for improvement of health outcomes and mitigation of the social effects of NCDs through the wider implementation of these resource-oriented community-based interventions is promising. Such approaches have the potential for long-term sustainability, as they rely on existing social networks, traditional medicine interventions and community assets rather than external funding sources or healthcare infrastructure.^7^ Furthermore, indigenous and other vulnerable populations often face additional barriers (eg, discrimination) and may therefore benefit the most from this kind of intervention.^8^

Despite the evidence from high-income countries,^4 9 10^ the availability and effectiveness of resource-oriented interventions in indigenous and vulnerable populations in Latin America, specifically in Bolivia, Colombia and Guatemala, has not been fully addressed. It is imperative when designing culturally sensitive and context-appropriate interventions to first explore local needs. This will allow interventions to use and build on the capacities of existing institutions to support community action.

A situation analysis (SA) is a flexible approach to comprehensively examine a specific health context, whether a country, a region or a health facility. It makes use of multiple research methodologies, as either a single, multiple or formal mixed-method approach, in an agile, pragmatic and rigorous way.^11 12^ The overall goal of a SA is to facilitate planning, implementation and research into public policies, plans, programmes or individual interventions^11 12^ aiming to improve healthcare and reduce the burden of NCDs. We conducted a SA that aimed to (1) describe the current health situation regarding common physical and mental NCDs, (2) describe the current availability of community-based services and (3) understand barriers and facilitators to community-based care within three regions of Bolivia, Colombia and Guatemala.

## Methods

### Study design and participants

Multiple methods of data collection were applied to obtain the views of different stakeholders, including health workers, community leads, patients and caregivers. Our SA spanned specific regions and health centres (HCs) in Bolivia, Colombia and Guatemala, employing quantitative and qualitative approaches. For the quantitative dimension, we reviewed secondary databases to identify the main social and health indicators in each country, region and HC. Concurrently, we collected additional HC-specific quantitative data using a survey. This was supplemented with qualitative data obtained from semistructured interviews. This multifaceted approach enabled us to understand the context for the community management of NCDs. This study was conducted and reported according to the Strengthening the Reporting of Observational Studies in Epidemiology checklist for cross-sectional studies and the Standards for Reporting Qualitative Research checklist for qualitative studies.^13 14^

### Setting and country description

**Figure 1:**
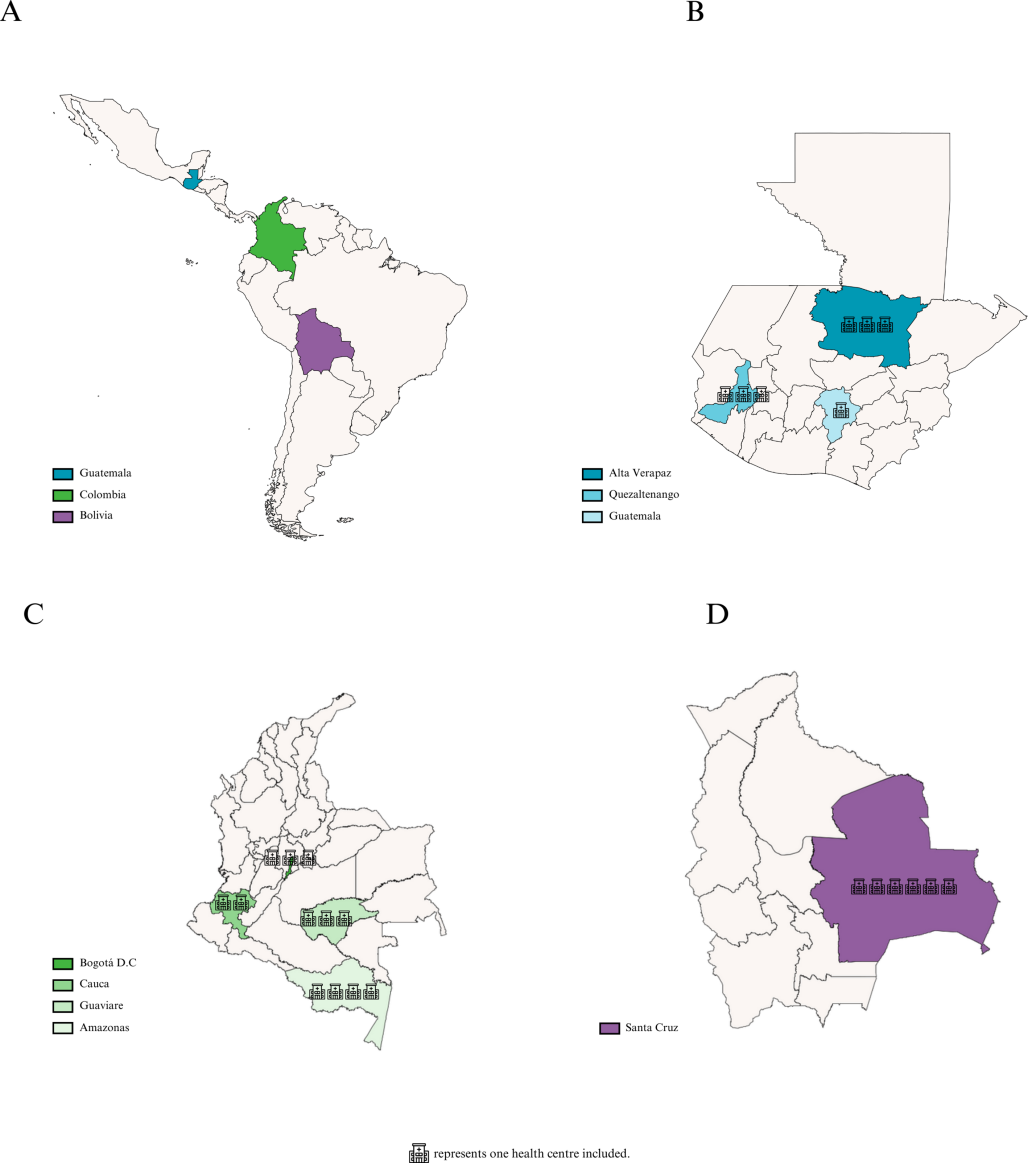
Countries, regions and health centres included in the situational analysis.

The study took place in Santa Cruz de la Sierra, Bolivia, which includes both urban and rural regions. In Colombia, the departments of Amazonas, Guaviare, Cauca and Bogotá (capital district) were included. In Guatemala, three regions were chosen: Quetzaltenango, Alta Verapaz and Guatemala Central. The countries, regions and centres included are represented in figure 1.

### Participants

We recruited two participant groups for this study: healthcare centre representatives for the survey component and multiple stakeholders for the qualitative interviews.

For the survey, the lead clinician or service manager from each HC was invited to participate. Eligibility criteria included (1) experience in managing patients with common NCDs, such as diabetes, obesity, hypertension, chronic obstructive pulmonary disease (COPD), asthma, anxiety, depression or alcohol misuse and (2) knowledge of institutional functioning, including available services, interventions and community health strategies.

For the qualitative semistructured interviews, purposive sampling was used to recruit up to two participants from each of four stakeholder groups per HC: healthcare practitioners (HCPs), community health workers (CHWs), patients and carers or caregivers. Patients were required to have a diagnosis of one or more of the listed conditions and to be currently receiving care at a participating HC. Caregivers were required to actively support patients with these conditions. HCPs were required to have experience treating affected patients, and CHWs were required to be actively engaged with communities served by the participating HC. All participants were aged 18 years or older and provided informed consent.

### Measurement set

For the survey, a predesigned structured questionnaire used in a previous SA^15^ was translated into Latin-American Spanish, adapted for the current study objectives and adjusted to the cultural needs of each country. The tool captured data on (1) the structure of local services, (2) healthcare information systems, (3) the number and characteristics of patients who use the service, (4) the number and qualifications of employed medical professionals, (5) patient care pathways, (6) components of routine care, (7) infrastructure, (8) the current use of technological tools and readiness to adopt new ones, (9) capacities and challenges in providing continuous training for healthcare personnel, (10) the extent to which patients are empowered in healthcare and (11) quantitative data on the prevalence of relevant health conditions.

The guides for the semistructured interviews were developed based on the questionnaire. Adaptations were made for each participant type, including healthcare professionals, patients, carers and CHWs. The interviews addressed the following topics: (1) the organisational structure, service delivery and information systems; (2) the barriers and challenges in accessing and delivering community-based care; (3) the current use of technological tools and readiness to adopt new ones; (4) the capacities and barriers in providing continuous training for healthcare personnel and (5) the empowerment of users in healthcare.

### Procedures

The national information systems of each country were utilised to gather sociodemographic indicators. These indicators include the general population, the population over 18 years of age, the self-identified Indigenous population, life expectancy, birth rate, crude mortality rate and coverage of essential services. We searched for each indicator at a national and regional level. We also calculated the prevalence of diabetes, obesity, hypertension, COPD, asthma, anxiety, depression and alcohol consumption-related health problems at national, regional and HC levels. National and regional information was obtained from records available in the information systems in each country, and information related to health facilities was identified according to health facility records. We collected the total number of patients with each diagnosis during each year (2018- 2022) and/or the total number of appointments related to each diagnosis per year. Diagnoses were established according to the International Statistical Classification of Diseases and Related Health Problems 10th Revision.^16^ We also identified the total number of patients or appointments for every analysis level.

HCs were identified based on the knowledge and experience of the research team within the communities and via local networks. After identifying a HC, the selected clinician or service manager was contacted to fill out the survey. The survey was administered by a research team member after obtaining informed consent.

Key HCPs within each HC were identified using inperson snowballing techniques. Initially, the lead clinicians contributed to identifying potentially eligible staff members. Following the initial clinician interviews, either the staff or the site leader aided in identifying patients, carers and community healthcare workers. After obtaining informed consent, trained researchers conducted semistructured interviews in Spanish in a quiet, private space. All interviews were audio-recorded and transcribed verbatim. The interviewers underwent prior training in qualitative methodologies and data collection techniques.

### Data analysis

To describe the current health situation regarding common physical and mental NCDs, the sociodemographic indicators were obtained using the available data in each country and region. For the health indicators, we defined prevalence as the proportion of patients receiving treatment for each diagnosis relative to the total number of patients cared for due to any cause. Alternatively, prevalence was defined as the proportion of appointments held for a specific diagnosis relative to the total number of appointments for any cause. Prevalence proportions were computed for each diagnosis and year at each level of analysis (national, regional and HC) and presented per 1000 population.

To describe the current availability of community-based services and understand barriers and facilitators of community-based care, the survey was summarised in a matrix for each HC. Interviews were analysed using thematic framework analysis.^17^ The individual interviews were first transcribed verbatim employing Whisper AI^18^ and then reviewed manually. Prior to sharing with the research team, all interviews were anonymised. The topic guide was used as an initial guide to develop themes and codes. Following data immersion, two interviews were coded per region per country, and the research team agreed on a joint codebook. Researchers adjusted the codebook iteratively; minimum changes were made after the initial consensus. All interviews were coded using QSR NVivo V.14.

Quantitative and qualitative data were not triangulated. Nevertheless, data provide a comprehensive description of NCD morbidity in the countries and regions where the HCs are located. Also, the local morbidity data in each HC and the current state of services were assessed. This information fulfilled the purpose of supporting decision-making in favour of implementing community-based strategies for managing NCDs.

### Patient and public involvement

Members of the participant communities were involved as research field assistants for data collection. Communities and the participating HC were also involved in disseminating the results. We employed different methodologies adapted to the cultural context of the communities. Health authorities from the HC were also informed of the results. For example, we shared a calendar with relevant information from the results. Before sharing the product with the community, input was sought from community leaders to ensure comprehension and relevance. Second, data analysis, making use of the data collected, will be published in local journals with the participation of community research field assistants as coauthors.

## Results

**Figure 2:**
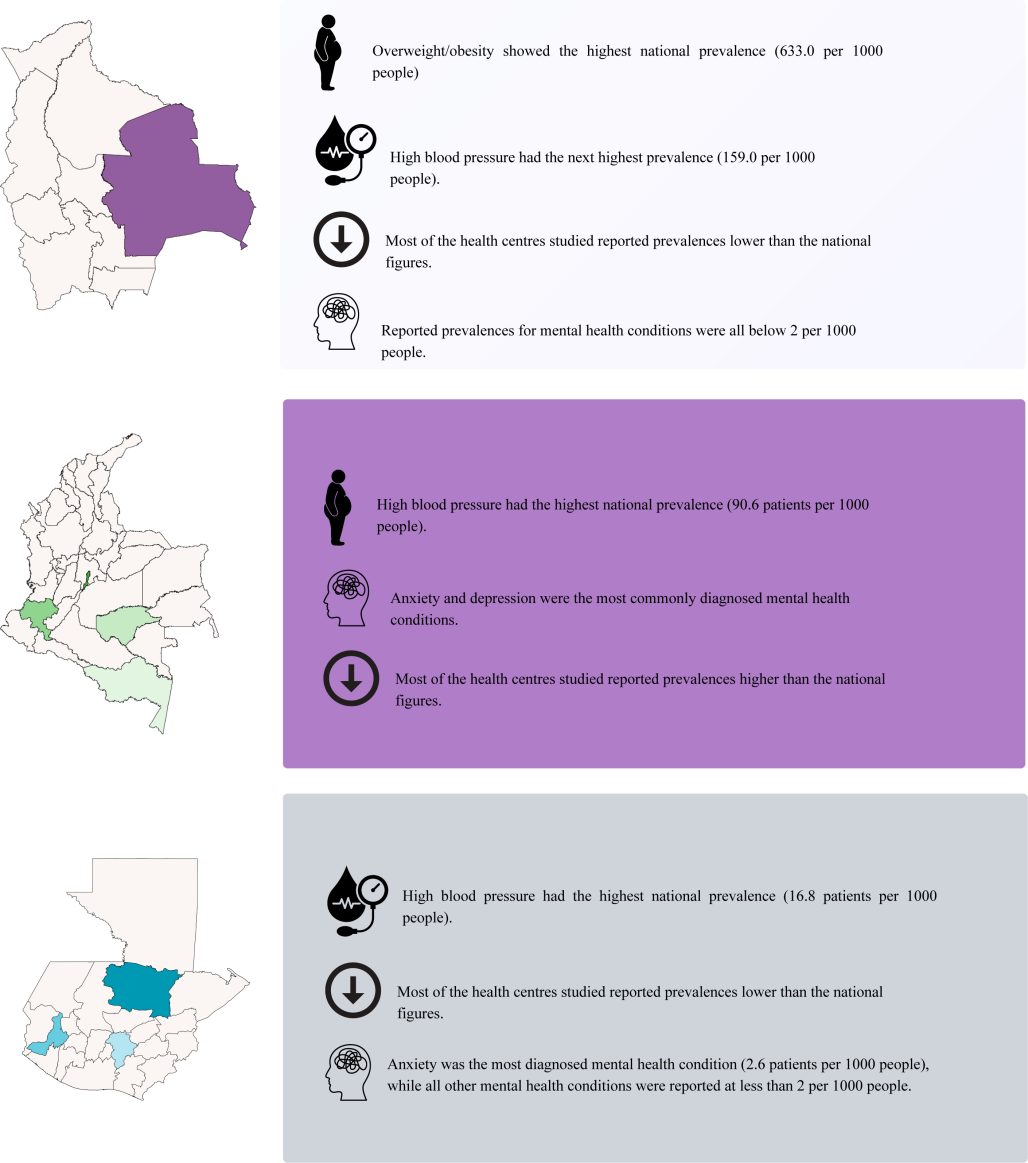
Overview of main study results.

Sociodemographic indicators collected as contextual information from each country and region are detailed in online supplemental material 1. Additionally, figure 2 graphically depicts the main study results.

25 HCs were included, and the distribution by country and region can be found in figure 1. The number of surveys and interviews conducted in each country according to the type of participant is described in table 1. Of the 108 participants, 25% were HC leaders, 25% health professionals, 22% patients, 12% caregivers and 16% CHWs.

**Table 1:** Number of surveys and interviews per country and type of participant

Country	Surveys	HCPs	Patients	Carers	CHWs
		Interviews	Interviews	Interviews	Interviews
Bolivia	13	17	10	4	3
Colombia	6	6	8	7	12
Guatemala	8	4	6	2	2

CHW, community health worker; HCP, healthcare practitioners.

### Description of the characteristics of the included HCs

Table 2 provides an overview of HCs characteristics in the three countries, emphasising aspects such as HC complexity, the nature of health records and the range of services offered. HCs in Bolivia ranged from low to high complexity, with most relying on paper-based records, reflecting the infrastructural challenges faced in some regions. In contrast, Colombia’s HCs were more likely to use electronic health records, particularly in urban settings, where there was a higher demand. Guatemala, similar to Bolivia, was predominantly paper-based but did report high daily patient volumes, especially for mental health patients.

**Table 2:** Overview of health centre characteristics

Country (health centre)	Complexity level	Health records	Setting	Available HS	Daily patients PH§	Daily patients MH*	Healthcare practitioners†	Appointment duration‡
Bolivia (A)	Low	Paper	Urban	PH	208	–	–	15
Bolivia (B)	Low	Electronic and paper	Urban	PH	204	–	81	15
Bolivia (C)	Low	Paper	Urban	PH and MH	4	–	43	20–60
Bolivia (D)	High	Paper	Urban	MH	–	46	52	–
Bolivia (E)	Medium	Electronic and paper	Urban	PH and MH	270	10	300	15–20
Bolivia (F)	Medium	Paper	Urban	PH and MH	–	–	–	15–20
Colombia (G)	Medium	Electronic	Urban	PH and MH	16	4	28	30–60
Colombia (H)	Low	Electronic and paper	Urban	PH and MH	16	13	18	20–40
Colombia (I)	Low	Electronic	Urban	MH	–	40	9	30–40
Colombia (J)	Medium	Electronic	Urban	PH and MH	63	7	27	30–40
Colombia (K)	Low	Electronic and paper	Urban	PH and MH	57	15	22	20–30
Colombia (L)	Medium	Electronic and paper	Urban	MH	0	84	18	30
Colombia (M)	Low	Electronic and paper	Urban	PH and MH	10	10	8	20–30
Colombia (N)	Low	Electronic and paper	Urban	PH and MH	–	–	80	45
Colombia (O)	Low	Electronic and paper	Urban	PH and MH	–	–	120	45
Colombia (P1)	Medium	Electronic and paper	Urban	PH and MH	–	–	350	20–30
Colombia (P2)	Low	Electronic	Rural	PH and MH	3	1	2	30–60
Colombia (P3)	Low	Electronic	Rural	PH and MH	8	–	2	30–60
Guatemala (Q)	Medium	Paper	Urban	PH and MH	17	0	112	20–90
Guatemala (R)	Medium	Paper	Urban	PH and MH	4	–	172	30–90
Guatemala (S)	Medium	Paper	Urban	PH and MH	21	<1	40	15–60
Guatemala (T)	Low	Electronic and paper	Rural	PH and MH	0	–	6	20–30
Guatemala (U)	Medium	Electronic and paper	Urban	PH and MH	4	–	64	30
Guatemala (V)	High	Electronic and paper	Urban	PH and MH	12	57	86	20–30
Guatemala (W)	Medium	Electronic	Urban	PH and MH	69	1	59	20–40

* Average number of patients with mental health conditions seen in each centre reported by health leaders.

† Approximate number of healthcare practitioners serving in each centre reported by health leaders.

‡ Estimated appointment duration reported in minutes for each health centre.

§ Average number of patients with non-communicable diseases seen in each centre reported by health leaders.

HS, health services; MH, mental health; PH, physical health.

The table further indicates that staffing levels and appointment duration varied across HCs, with Bolivia showing relatively modest figures for healthcare professionals compared with Colombia, where urban centres had significantly higher staffing levels and appointment throughput.

The distribution of healthcare personnel across the three countries is presented in online supplemental material 2, highlighting the number and types of healthcare workers at each HC. In Bolivia, HCs were predominantly staffed with nursing assistants and registered nurses, with limited numbers of specialists such as dieticians or psychologists. Colombia’s HC P1 was the most notable for its large number of general practitioners. Conversely, Guatemala reported lower staffing levels, with many HCs lacking specialists such as psychologists or dietitians.

### Prevalences of health conditions

At a national level, Bolivia’s prevalence of overweight individuals and obesity was the highest for any of the countries and conditions (633.0 per 1000 people). After this peak, hypertension was the most prevalent condition in 2022, with figures of 159.0, 90.6 and 116.8 per 1000 for Bolivia, Colombia and Guatemala, respectively. Mental health conditions were less frequent across all countries and regions studied, with anxiety and depression being the most prevalent among this group of conditions (table 3).

**Table 3:** Prevalence proportions of NCDs and mental health conditions in 2022 (per 1000 people)

Region (country)	Diabetes	Overweight and obesity	HBP	Asthma	COPD	Depression	Anxiety	Alcohol-related diseases
City of Santa Cruz* (Bolivia)	14.7	5.8	21.0	2.4	0.4	0.5	1.9	0.1
City of San José de Chiquitos* (Bolivia)	10.1	11.1	20.4	0.5	0.1	0.0	0.0	0.0
Department of Santa Cruz* (Bolivia)	14.5	4.1	23.3	2.0	0.3	0.3	1.3	0.1
National†(Bolivia)	37.0	633.0	159.0	1.2	0.2	0.4	1.2	0.2
Amazonas (Colombia‡)	11.3	6.8	30.3	1.7	0.5	1.8	3.4	1.4
Bogotá D.C (Colombia‡)	31.4	24.0	96.6	6.1	1.3	8.1	16.4	0.7
Cauca (Colombia‡)	22.2	10.9	71.7	2.3	0.5	2.2	7.1	0.6
Guaviare (Colombia‡)	24.8	14.8	69.4	2.9	0.5	2.8	4.7	0.9
National (Colombia‡)	26.6	15.2	90.6	3.6	0.9	5.1	12.2	0.6
Alta Verapaz (Guatemala§)	6.6	0.6	10.0	1.2	0.1	0.2	1.0	0.0
Central Guatemala (Guatemala§)	7.3	1.6	10.3	0.5	0.1	1.2	2.4	0.2
Quetzaltenango (Guatemala§)	9.0	2.1	10.2	0.6	0.8	0.5	3.7	0.9
National (Guatemala§)	13.9	1.4	16.8	1.2	0.2	0.7	2.6	0.7

* Data source: the prevalence calculations were based on data from the National Epidemiological Surveillance System of Bolivia.

† Data on the prevalence of diabetes, overweight and obesity and high blood pressure are available up to 2021. The National Survey on Non-communicable Diseases was the source of these data.

‡ Data source: the prevalence calculations were based on data from the Health Services Information System and the National Administrative Department of Statistics of Colombia.

§ Data source: the prevalence calculations were based on data from the Information Technologies Direction· Ministry of Public Health and Social Assistance of the Republic of Guatemala. ICD10 codes: diabetes (E10-E14), obesity (E66), HPB (I10), asthma (J45), COPD (J44), depression (F32, F33), anxiety (F40, F41), bipolar disorder (F31), schizophrenia (F20), alcohol-related diseases (F10).

COPD, chronic obstructive pulmonary disease; HPB, high blood pressure ; ICD10, International Statistical Classification of Diseases and Related Health Problems 10th Revision; NCDs, non-communicable diseases.

Table 4 and online supplemental table S3 examine the prevalence of key health conditions at the HC level. Notably, the HC F in Bolivia reported a high rate of diabetes (169.8 per 1000 people), while the HC L in Colombia, which specialises in mental health conditions healthcare, had particularly high rates of schizophrenia (432.8 per 1000 people) and bipolar disorder (300.6 per 1000 people). Among Guatemala’s centres, the most prevalent condition reported was hypertension (9.7 per 1000 people in the HC Q).

**Table 4:** Health centres prevalence proportions for NCDs and mental health conditions (per 1000 people)

Country (health centre)	Diabetes	Overweight and obesity	HBP	Asthma	COPD	Depression	Anxiety	Alcohol-related diseases
Bolivia* (A)	103.5	4.1	76.7	0.2	0.2	0.2	0.0	0.2
Bolivia* (B)	38.0	1.1	47.6	3.0	0.0	†	0.7	†
Bolivia* (C)	†	†	†	†	†	†	†	†
Bolivia* (D)	†	†	†	†	†	136.4	168.3	13.7
Bolivia* (E)	69.8	3.2	104.3	5.1	5.2	2.4	2.0	0.2
Bolivia* (F)	169.8	†	267.3	8.4	†	†	†	†
Colombia‡ (G)	36.7	21.9	107.5	2.5	0.8	0.4	10.1	1.7
Colombia‡ (H)	59.7		239.4	8.3	4.1	21.5	1.7	1.7
Colombia‡ (I)	†	†	†	†	†	18.9	0.0	18.9
Colombia‡ (J)	48.1	57.5	3.4	19.1	7.8	0.8	0.2	3.8
Colombia‡ (K)	49.3	62.3	233.2	7.8	3.2	5.5	40.0	0.7
Colombia‡ (L)	9.8	6.8	40.0	2.3	0.8	154.8	183.5	9.8
Colombia‡ (M)	41.6	11.3	178.9	4.4	2.2	23.2	32.8	0.7
Colombia‡ (N)	30.8	21.6	70.1	3.6	1.1	0.5	7.3	0.5
Colombia‡ (O)	54.0	22.8	81.4	3.0	0.8	2.6	7.2	1.2
Colombia‡ (P)	33.1	22.2	82.2	3.6	0.3	1.9	3.0	1.5
Guatemala§ (Q)	4.2	†	9.7	†	†	†	†	†
Guatemala§ (R)	1.3	0.3	4.2	0.6	0.0	0.0	0.1	0.0
Guatemala§ (S)	1.4	†	2.7	†	†	†	†	†
Guatemala§ (T)	†	†	†	†	†	†	†	†
Guatemala§ (U)	3.7	0.1	0.2	0.0	0.0	0.6	0.3	0.0
Guatemala§ (V)	1.0	0.8	2.3	†	†	2.5	2.5	0.1

* Data source: The prevalence calculations were based on the record of consultations by diagnosis among the total consultations obtained at the same health centre or through each centre’s network for 2022. For centre B, the availability of information on the prevalence of anxiety is up to the year 2021.

† Missing data were either not reported or not found in the reviewed data sources.

‡ Data source: The prevalence calculations were based on data from the health centres for 2023 except for centre P (2022 data).

§ Data source: The prevalence calculations of diabetes, HBP and alcohol-related diseases were based on data of vital statistics from Alta Verapaz and Quetzaltenango areas, 2022 · Ministry of Public Health and Social Assistance (MSPAS). The rest of disease cases were based on data from the health centres through surveys.

COPD, Chronic Obstructive Pulmonary Disease; HPB, High Blood Pressure; NCDs, non-communicable diseases.

### Current health practices: barriers and facilitators (Health institutions and communities)

To assess the current availability of community-based services and understand barriers and facilitators to community-based care, we conducted semistructured interviews. Data were classified into two main categories. The community interventions category refers to the services a HC provides to the community, whereas the community strategies category addresses the mechanisms that emerge within a community to promote its health and well-being.

### Community interventions

#### Operation of healthcare centres

All three countries have their own standardised long-term NCD programmes, which included mental health conditions. Follow-up of patients with NCDs in all centres usually occurs after one to 3 months, depending on how stable the pathology is and the patient’s adherence to treatment.

In all three countries, healthcare procedures are determined by protocols set by the respective ministries of health. These protocols include booking appointments, patient registration, taking vital signs and medical attention by healthcare professionals. As for the appointment scheduling process, a number of differences were identified among the countries. In some centres, patients can schedule and authorise their appointments online or in person.

(…) you need to get up early the day before so they’ll see you, and if you’re really sick, like this patient who can barely walk, and there’s no one to give you a ride, you’ll just die at home. Too much protocol! Too much bureaucracy to get the medical attention you really need. Caregiver, Bolivia.

Regardless of the process used to book appointments, patients in all three countries perceive limitations to accessing healthcare. In Colombia and Bolivia, patients must go through different administrative processes, while in some Guatemalan HCs, they must show up in person, not knowing with certainty whether they will be seen. Moreover, in all three countries, qualitative data revealed issues regarding the availability of healthcare professionals.

Long waiting times and paperwork discourage patients from attending treatment, with some of the interviewees highlighting how time is a barrier to treatment. In addition to the difficulties in getting appointments, limitations were identified in Bolivia and Colombia regarding the limited duration and low frequency of the appointments. This was in contrast to what healthcare professionals in Guatemala expressed.

If you’re not that sick when you reach San José [del Guaviare], you’re not going to get an appointment. You must make an outpatient appointment, so you need to go, get a token, queue up for a longtime, get up early and see if you can get a token. Once you get the token, they’ll tell you whether they can see you or if you have to come back the next day. After that, you have to wait for the doctor to see you. If they refer you to an internist, you need to go back to the IPS to get an authorisation, which takes from one to two weeks. Community leader, Colombia.

[Patients] do not always have that time available due to work or other commitments. They have a hard time stepping away from their duties for an hour, hour and a half, to come here. Why an hour and a half? More or less; people usually walk to the medical centr. Sometimes they ride the bus or a pick-up truck, but most of them walk here, so they need to take more time off. Healthcare professional, Guatemala.

Most of the interviews mentioned the presence of nurses and general practitioners and, sometimes, psychologists, therapists, dentists and healthcare professionals in training. However, patients and caregivers feel there is a shortage of specialised healthcare workers and services. In particular, mental healthcare was seen as limited with a lack of training on this topic reported by healthcare managers and staff. Furthermore, there is a need for administrative staff, as in many cases, healthcare professionals must assume this task, taking away from clinical care. In Bolivia and Colombia, patients note that the high healthcare turnover and the lack of continuity in treatment are limitations to adequate healthcare.

It’s an issue I’ve always mentioned; in the future, this will be a public health issue, […], which is the area of mental health. It’s an issue that, for the moment, hasn’t been deeply addressed within the institution, at the national level, or globally […] What do I mean by this? I mean that mental illnesses or disorders are not taken as seriously as should be taken. Healthcare professional, Colombia.

Well, in this case, here or in other medical centres, those who see the patients are not licensed doctors, they’re interns. When there are no interns, the professional [nurse] handles the consultation, in addition to anything related to the job and all the administrative work. And the assistant… when the professional is not in, the assistant must do all the work, including consultations. Healthcare professional, Guatemala.

They came (…) an excellent lady (…) but she left, she didn’t stay long; they don’t stick around, sometimes they come without an ‘indefinite-term contract,’ they need to find room and board themselves and, you know, everything’s about money, now… they leave, and we’re left with the same people as before. Patient, Bolivia.

HCs in the three countries provide patient care through health campaigns or brigades seeking to offer medical services not usually available at the clinics. However, these services are faced with multiple difficulties in regions such as Guaviare (Colombia), where healthcare professionals believe these brigades are not enough, as they do not fully address the needs of the community. In San José de Chiquitos (Bolivia), the healthcare promotion and prevention brigades are widely accepted; however, such activities in other Bolivian centres are usually scarce.

Oh, Jesus! We need many more doctors, more patient accessibility to places, especially when I go to the provinces and I see that… it breaks my heart. To get to where we’re having the brigade, they walk an hour or two, thanking God the doctor’s here. Healthcare professional, Guatemala.

Yeah, I… that’s why I want them to bring someone [psychologists] to the brigades, so that the people I’ve already identified can vent and figure out what to do. Healthcare professional, Colombia.

No. Only on vaccination campaigns… they go door to door, sometimes, and sometimes they come to a place in a neighborhood, to administer vaccines… but they don’t do house calls, not even during COVID! (…) No! Patient, Bolivia.

Colombia and Guatemala offer house calls to elderly patients and indigenous communities in rural areas. In Guatemala, this type of care is also available to pregnant women and is very well perceived by caregivers and patients. In the three countries, they also receive education about the disease and treatment. Likewise, caregivers usually attend group activities with the patient, including workshops for long-term patients. Finally, patients feel that health personnel are usually empathetic in their care, which promotes an adequate doctor-patient relationship.

They’ve also done house calls for women who have recently given birth and can’t leave their homes. Doctors and a nurse see them at home, but that mostly happens in small towns. Healthcare professional, Guatemala.

The diagnosis is explained to caregivers or companions, especially if they are family, because they are made aware of the hereditary factor, family diet and the check-ups they need. And that they should join the ‘Diabetic Patient Club.’ Healthcare professional, Guatemala.

I’ve heard many things about the floor attendant [nurse], there are several complaints about him. They say things like: ‘you go there and he’s such a grump.’ Maybe that’s why… People are very confrontational. They’re never satisfied with anything. They know there’s a doctor here and they don’t come. Look, it’s Tuesday, and there are no consultations. Healthcare professional, Colombia.

#### Infrastructure

Patients and healthcare workers from all three countries believe that long distances to HCs and poor access roads are compounded by economic hardship in the communities. For instance, in Guatemala, when there are programmes in place for monitoring pathologies such as diabetes, patients prefer not to attend due to these limitations.

If I have no means to move, I’d rather stay at home and accept God’s will. Sometimes we can leave, but I need at least 10 quetzales [US$1.30] to go downtown and back. But, if I don’t have that money? I can’t tell the driver ‘take me there and I’ll pay you later’ [laughs]. That’s why you don’t go, despite not feeling well. Patient, Guatemala.

The inadequacy of some of the physical spaces used to provide the services was highlighted. For instance, a shortage of medical equipment and a lack of essential services such as water supply in some rural areas was reported. In Guaviare, medical centres do not have some basic medical instruments, and access to medicine is limited. Furthermore, these spaces do not always ensure patient privacy.

There are probably more issues with medicines, lack of medicine and supplies. That’s the main thing, because you’ll come here as a patient and they’ll tell you they can provide you with all the care, but then they don’t even have acetaminophen. We can’t do this without supplies! Healthcare professional, Guatemala.

In terms of technological infrastructure, HCs in Colombia and Guatemala use electronic medical records. However, healthcare workers in all three countries point out some drawbacks, emphasising the lack of computer equipment available, poor training and skills in technology, limited consultation time to implement technological tools, poor internet connection and intermittent electricity service, particularly in some areas in Guatemala. Despite these challenges, healthcare professionals were perceived to be willing to use technology in healthcare.

I think it would be a matter of getting used to it, let’s say, wouldn't it? I think it would be very useful, to be honest, the question of technology, (…) our generation does not have much difficulty with technology yet, so we understand it, don’t we? Healthcare professional, Bolivia

#### Culture

In Guatemala, healthcare professionals highlight the prevalence of sexism in rural areas. For example, women require authorisation from their husbands to receive medical care. Furthermore, the healthcare professionals in the three countries felt that the community stigmatises mental health, which contributes to low adherence to these services.

Women need permission to go to the doctor. So, for example, if the husband disagrees with her coming in twice or once a week because she’s neglecting the house, the kids, and him, she can’t go. And that’s when I have trouble treating them more often. Healthcare professional, Guatemala.

Talking about mental health here is taboo. Yeah, people have trouble talking about this. And they may have problems, but won’t ever talk about them. They’re very closed-minded. I don’t know if it’s because of the environment where they are raised, but talking about mental health—no, they don’t even believe in it. Healthcare professional, Colombia.

The healthcare professionals in all three countries report low adherence and acceptance of treatments, recommendations and suggestions made by health workers. To counteract this, professionals from the three countries focus on providing education and emphasising the importance of treatment.

The family and the community always interfere. They’d rather go to the *comadre*, the folk healer, the church they go to. When they’ve run out of all the family resources and see no results, that’s when they come. Healthcare professional, Bolivia.

Seriously, people are so lazy! They don’t care about their health. Here, I tell all patients that they should at least come for a check-up once or twice a year. CHW, Bolivia.

In Guatemala, several patients report that the population faces difficulties in expressing their symptoms or understanding the professionals. This is because healthcare professionals do not speak the local languages and, in many cases, lack knowledge of natural medicines, which makes patients feel discriminated against when they mention them. Despite this, in all three countries, the administration of each HC is concerned with improving the provision of services from an intercultural perspective in which traditional and Western medical knowledge complement each other.

Those who come here should be kind enough to speak a little bit of K’iche’*. It’s necessary, and the truth is that there are many people with different ailments they can’t explain, but not because they don’t have the courage to speak up… they don’t have the means to explain their symptoms. Patient, Guatemala.

K’iche’ * One of Guatemala’s 24 indigenous languages.

### Community strategies

#### Self-care practices

In Colombia and Guatemala, perceptions are shared about the importance of community practices associated with good nutrition, diverse diets and access to adequate services such as drinking water. Communities in the three countries have adopted care practices related to the transformation and consumption of natural plants at home to combat and prevent NCDs.

Grapefruit is very good for blood pressure, or tangerine that is still a little green. (…) It regulates blood pressure, and I say so because it regulated mine. For high blood pressure, it’s the guava plant, the *sabanera* guava. You take three buds of the *sabanera* guava and cook them in a glass of water, let it rest, drink it warm, not too hot or cold, and you’ll be okay really quick. Community worker, Colombia.

Well, the medicine we’ve discovered here is useful for many things, especially for coughs. It’s also useful for lower abdominal pain, especially for women, and many other things. Others grind it… they drink the water for diabetes, which helps a lot because, during coronavirus, that’s what most of the community fought it with. CHW, Bolivia.

#### Community actions and services

In both Bolivia and Colombia, solidarity and intercultural health practices are carried out to serve remote areas. These processes include resilience, adaptation and rapid response strategies of communities to care for patients with serious or urgent health conditions, such as contracting emergency services and logistical support to schedule medical appointments. These support networks and healthcare models are suited to the conditions and resources available in each community.

(…) same thing here… most people (…) are neighbors, and they help each other (…) we can help patients and get them to a place where they have been cared for in a helpful way. Community worker, Bolivia.

In Colombia, complex and organised systems of communication between indigenous health authorities, Peace Signatories and farmers with HCs and hospitals are mentioned. This facilitates patient care, in addition to fostering an intercultural dialogue of knowledge about health. Ancestral and alternative practices are combined and articulated with knowledge and treatments from conventional or Western medicine. For instance, in Colombia and Bolivia, some community workers shared the existence of indigenous associations that work with plants and produce medicines for their communities.

Leaders are in contact with health entities themselves; for instance, in a case where the nurses are in referral over there with another patient. So I said: ‘well, no, sir, the thing is that we have a patient here who just came in, so we need to solve this case immediately, because it’s a serious matter.’ And then, they said: ‘okay, we’ll be ready at this time’… Community worker, Colombia

Finally, there are people from the communities in Colombia and Bolivia who undergo academic and empirical training and return to their hometowns, joining the local health systems. This situation is perceived as a strength for their improvement. There are also community workers (Bolivia), midwives (Guatemala) and massage therapists who are part of health committees and local authorities (Colombia) who act as a bridge between the health centres and the communities where they live.

… When I started making syrups to cure children, which is not available in academic medicine… and making remedies for different diseases. I’ve been representing this project for the past 5 years. As a group facilitator I have learned more… CHW, Bolivia.

## Discussion

Our results revealed a high burden of diabetes, hypertension and individuals classed as overweight, as well as overall low figures for mental health conditions, except for some HCs, particularly in Bolivia and Colombia. We also identified some differences between urban and rural areas. In the HCs, we identified particular infrastructure and health personnel’s capabilities for community care. Also, some local health programmes anchored to the national level health system support the management of NCDs. It was also possible to identify health models designed to fulfil the particular ethnic and cultural aspects of health in some communities. However, we also identified several areas requiring improvement, including barriers to culturally appropriate care, structural limitations within the health system and challenges in NCD data management.

The findings highlight significant challenges in addressing NCDs. One of the major limitations identified is the lack of comparability in data due to varying types of records and data collection methods across the countries. Also, as previously reported in other LMICs,^19^ under-reporting may be another limitation that contributes to explaining the disparities, for example, between local and national prevalences in Bolivia. The disparity between Bolivia’s national and regional data suggests that the regional prevalences are probably underestimated. While the national data came from a national survey based on a representative sample of the population, the regional data source was a report based on the reasons for consultation and not the precise diagnosis of the patient. In Colombia, data were more consistent, but underestimation can compromise the results due to the sole registry of the first diagnosis, leaving comorbidities out of sight. These issues substantially impact research and healthcare policy, underscoring the urgent need for initiatives that seek standardised, high-quality data collection and record-keeping practices. A unified approach would greatly enhance data reliability and comparability, allowing for more accurate assessments of NCD prevalence and more informed decision-making.^20^

Moreover, several structural and systemic barriers hinder the implementation of effective community healthcare interventions. For instance, indigenous healthcare models have not been adequately evaluated regarding their effectiveness, limiting their practical application. Additionally, as reported in Ghana, high staff turnover, a shortage of personnel, limited resources and logistical challenges such as long distances to healthcare facilities further impede the provision of care and future community-based health programmes.^21^ These barriers are particularly problematic in rural and remote areas where access to health services is already limited.

The relatively few mental health professionals (psychologists and psychiatrists), along with the perceptions of patients and healthcare professionals, place mental health as a significant concern. This is confounded by limitations faced regarding the availability of specialised services, trained professionals and community strategies in line with prior reports in Colombia and other LMICs.^22 23^ Mental health stigma further increases these limitations, as identified by HCPs and previously reported in some African and Asian countries. Stigma requires action to enhance health services use, adherence to treatment and support for people living with mental health conditions.^23^ As opposed to previous findings in LMICs relating to community healthcare strategies, we identified existing mechanisms through which the health system and traditional medicine interact, thus facilitating the future availability of community-based psychosocial interventions.^23^

Based on these findings, we recommend the establishment of unified data collection protocols to improve the quality and comparability of health information at the country level. High-quality data has the power to support health policy decision-making and enable more effective resource allocation.^24 25^ Efforts to strengthen data infrastructure should be complemented with investment in the training and retention of healthcare personnel, as a crucial endeavour to facilitate the continuity of care, which ultimately translates into better care.

Moreover, strategies such as enhancing mobile teams, establishing HCs in remote areas to reduce the population-to-facility ratio and improving road infrastructure could significantly enhance access to health services, particularly in underserved areas, thereby helping to mitigate the growing burden of NCDs in these regions. By addressing these systemic and infrastructural issues, it is possible to develop and implement more effective NCD prevention and management strategies in LMICs, such as community-based services that create positive health, social and economic impacts.^22^

Furthermore, based on the identified potential of indigenous healthcare models, we advocate for their wider involvement in health-related policy decision-making, recognising the potential of Indigenous communities to contribute to managing NCDs and by sharing knowledge with others.

### Strengths and limitations

The study had several strengths. First, including both mental and physical health facilities in the SA expanded the scope of the evaluation, facilitating the future implementation of integrated community-based strategies to comprehensively address NCDs. It also calls for solid integration at the health system level. Second, incorporating urban and rural HCs alongside HCPs from different backgrounds, patients, caregivers and CHWs has enabled us to obtain multiple perspectives on the barriers and facilitators to community care. Finally, the methodological strengths include the multi-method approach, which has provided a broader and valuable assessment of the strengths and weaknesses of community management of NCDs in Bolivia, Colombia and Guatemala.

However, it is important to highlight some limitations. As secondary data from national registers were used to present morbidity and social indicators, data quality relies on the original source, and some issues may cause an underestimation, particularly in the prevalence of diseases. Also, the last year of available information was from 2022 or 2023 and not necessarily the same across countries. Another drawback of the study is that the selection of the regions and HCs was not representative of any region or country. Therefore, results are focused on these specific communities where community-based interventions are being planned. A third limitation was the lack of involvement of cancer and maternal health. Both groups can help describe the capacity of a community health system, and extensive research in community management is needed, particularly in Latin America.^26 27^ Finally, although we included a wide range of voices from participants from different backgrounds and ethnic origins, we focused on the perception of different participants regarding the capacity of the HCs. Therefore, no sex-based or gender analysis was performed.

## Conclusion

Community-based interventions are powerful tools to support the management of long-term physical and mental health conditions in Latin America. This study presented barriers and facilitators for community care and discussed how addressing these barriers could strengthen healthcare systems and enhance the effectiveness of community-led strategies. This has important implications for future implementation research and for the development of sustainable and contextually appropriate interventions aimed at improving health outcomes and quality of life for people living with long-term NCDs in Bolivia, Colombia, Guatemala and beyond.

## Supplementary material

10.1136/bmjgh-2025-020466 online supplemental file 1

10.1136/bmjgh-2025-020466 online supplemental file 2

10.1136/bmjgh-2025-020466 online supplemental file 3

## Data Availability

Data are available upon reasonable request.
