# Supplementary material for: Understanding the community management of long-term physical and mental health conditions in Bolivia, Colombia and Guatemala: a situational analysis
Source: BMJ Glob Health. 2026 Mar 9;11(3):e020466. doi: 10.1136/bmjgh-2025-020466 (PMC12983732; doi:10.1136/bmjgh-2025-020466)
Supplement: online supplemental file 1 [file bmjgh-11-3-s001.pdf]

Supplementary Material 1

Contextual information: socio-demographic indicators

Socio-demographic indicators collected as contextual information from each country and region are detailed in supplementary material 1.

In 2018, 60.1 % of Bolivia's population were adults over 18 years of age, with a proportional distribution between both sexes. Additionally, 32.51 % of the national population lived in rural areas, and 41.75 % recognised themselves as belonging to an indigenous ethnic group. Life expectancy at birth stood at 74.4 years in 2022, and for the same year, the crude birth rate was 10.33 live births (LB) per 100,000 population, and the crude mortality rate was 2.87 per 1,000 population.

By 2023, 73.1 % of Colombia's population were adults over 18 years of age, of whom 52 % were men and 48 % were women. Additionally, 22.3 % lived in rural areas. Furthermore, 3.3 % of the national population is self-determined as Indigenous, and 5.8 % is part of the Black, Afro-Colombian, Raizal, or Palenquero population. Life expectancy was 77.2 years, and the crude birth rate was 12.1 LB per 100,000 population. Colombia's most recent crude mortality rate was reported for 2021, with a 9.0 per 1,000 population.

By 2022, 62 % of Guatemala's population was over 18 years of age, of which 52 % were female and 48 % male. Additionally, 53 % lived in rural areas, and 56.7 % recognised themselves as belonging to an ethnic group. Life expectancy was 73.6 years, the crude birth rate was 22 LB per 100,000 population, and the crude mortality rate was 5.5 per 1,000 population.

Table S1 presents data on the coverage of essential services (energy, gas, water supply, sewerage, and garbage collection) in different regions of the three countries. This table underscores significant disparities in service provision between urban and rural areas. For instance, Santa Cruz in Bolivia demonstrated near-complete service coverage, while Amazonas in Colombia exhibited notably lower service availability, particularly in rural regions. Guatemala’s rural areas, such as Alta Verapaz, similarly faced challenges in accessing basic infrastructure. While urban areas generally reported higher coverage of essential services, rural zones were marked by limited access to water supply, sewage treatment, and waste disposal, highlighting significant infrastructure gaps that directly impact healthcare delivery.

Table S1. Coverage of essential services by country and region

Country	Region	Year	Energy (%)	Gas (%)	Water Supply (%)	Sewerage (%)	Garbage Collection (%)
Bolivia*	Santa Cruz de la Sierra	2012	98·8	94·8	94·0	53·1	78·6
	San José de Chiquitos	2012	91·0	76·9	75·3	1·5	43·1
	Santa Cruz	2021	98·4	90·2	84·9	41·3	72·3
	National	2021	96·7	86·8	64·4	48·9	56·7
Colombia**	Amazonas	2022	83·2	0·0	43·3	42·3	53·3
	Bogotá	2022	100·0	93·5	99·6	99·1	99·6
	Cauca	2022	97·3	26·5	74·8	40·5	42·6
	Guaviare	2022	93·0	15·9	50·9	47·3	60·4
	National	2022	98·6	68·6	88·9	75·4	82·8
Guatemala***	Alta Verapaz±	2022	51·8	11·8	46·2	7·9	30·5
	Guatemala central± † ‡	2022	99·7	85·3	100·0	100·0	100·0
	Quetzaltenango± ‡	2022	96·9	45·7	95·6	45·5	22·9
	National± ‡	2022	89·9	48·2	79·4	42·1	59·1

**Data source: National Institute of Statistics - National Census of Population and Housing for 2012 data and Household Survey for 2021 data.*

***Data source: National Administrative Department of Statistics (DANE)· Quality of Life Statistics.*

**** Data sources: MSPAS: Labour report - Vital statistics, Secretariat of Planning and Programming of the Presidency, Population Census and National Employment and Income Survey..*

± The percentage coverage data for Gas service is from the year 2018, reported by the last available population census. The health system does not register this service.

† The percentage coverage data for Energy and Gas services are from the state department and not from the central area.

‡ The percentage for Water Supply, Sewerage and Garbage Collection services are from the year 2021.
