# Supplementary material for: Understanding the community management of long-term physical and mental health conditions in Bolivia, Colombia and Guatemala: a situational analysis
Source: BMJ Glob Health. 2026 Mar 9;11(3):e020466. doi: 10.1136/bmjgh-2025-020466 (PMC12983732; doi:10.1136/bmjgh-2025-020466)
Supplement: online supplemental file 2 [file bmjgh-11-3-s002.pdf]

**Supplementary material 2 .**

**Table S1. Approximate number of each type of health personnel at each centre.**

| Health Centres± | A | B | C | D | E | F | G | H | I | J | K | L | M | N | O | P1 | P2 | P3 | Q | R | S | T | U | V | W |
|---|---|---|---|---|---|---|---|---|---|---|---|---|---|---|---|---|---|---|---|---|---|---|---|---|---|
| Health Care Practitioners | Bolivia | | | | | | Colombia | | | | | | | | | | | | Guatemala | | | | | | |
| General Practitioners | 7 | 24 | 21 | .. | 34 | .. | 8 | 4 | .. | 6 | 5 | 2 | 2 | 8 | 8 | 45 | .. | 1 | 6 | 7 | 4 | .. | 6 | 9 | 45 † |
| Dieticians | .. | .. | .. | .. | .. | .. | .. | .. | .. | .. | .. | .. | .. | .. | .. | .. | .. | .. | .. | 1 | 1 | .. | 1 | 2 | 1 |
| Psychologists | .. | .. | .. | 3 | 1 | .. | 2 | 2 | 3 | 4 | 1 | 2 | 1 | 2 | 1 | 2 | .. | .. | 1 | 1 | 2 | 1 | 1 | 1 | .. |
| Respiratory therapist | .. | .. | .. | .. | 7 | .. | .. | .. | .. | 2 | .. | 2 | .. | 0 | 0 | .. | .. | .. | .. | .. | .. | .. | .. | .. | .. |
| Physical therapist | .. | .. | .. | .. | .. | .. | 1 | .. | 0 | 2 | .. | .. | .. | 0 | 0 | 3 | .. | .. | .. | .. | .. | .. | .. | 1 | .. |
| Social workers | .. | .. | .. | 2 | 3 | .. | 1 | 1 | 1 | 3 | .. | 1 | .. | 0 | 0 | 2 | .. | .. | .. | .. | 1 | .. | 1 | 2 | .. |
| Registered Nurses | 1 | .. | 2 | 3 | 131 | .. | 10 | 3 | 3 | 4 | 1 | 3 | 1 | 5 | 4 | P | .. | 1 | 11 | 17 | 8 | 1 | 19 | 24 | .. |
| Nursing assistants | 7 | 7 | 10 | 9 | 180 | .. | 12 | 6 | 0 | 6 | 9 | 6 | 1 | 66 | 103 | P | 1 | .. | 79 | 126 | 8 | 1 | 24 | 27 | .. |
| Laboratory technicians | .. | 14 | 4 | 1 | 8 | .. | .. | .. | .. | .. | .. | .. | .. | .. | .. | .. | .. | .. | 2 | 4 | 4 | 1 | 1 | 3 | 2 |
| Cardiologists | .. | .. | .. | .. | 4 | .. | .. | .. | .. | .. | .. | .. | .. | .. | .. | .. | .. | .. | .. | .. | .. | .. | .. | .. | .. |
| Pulmonologists | .. | .. | .. | .. | 1 | .. | .. | .. | .. | .. | .. | .. | .. | .. | .. | 1 | .. | .. | .. | .. | .. | .. | .. | .. | .. |
| Specialist in diabetes | .. | .. | .. | .. | 2 | .. | .. | .. | .. | .. | .. | .. | .. | .. | .. | .. | .. | .. | .. | .. | .. | .. | .. | .. | .. |
| Psychiatrists | .. | .. | .. | 6 | .. | .. | 0 | .. | 1 | 0 | .. | 3 | .. | .. | .. | .. | .. | .. | .. | .. | .. | .. | .. | 1 | .. |
| Ocupational Therapists | .. | .. | .. | .. | .. | .. | 1 | 1 | 1 | 0 | .. | .. | .. | .. | .. | .. | .. | .. | .. | .. | .. | .. | .. | .. | .. |
| Ophthalmologists | .. | .. | .. | .. | .. | .. | .. | .. | .. | .. | 4 | .. | .. | .. | .. | .. | .. | .. | .. | .. | .. | .. | .. | .. | .. |
| Ondontologists | .. | .. | 2 | 1 | .. | .. | .. | .. | .. | .. | 2 | .. | 2 | 4 | 4 | .. | .. | .. | .. | 1 | 1 | .. | .. | .. | 5 |

| | | | | | | | | | | | | | | | | | | | | | | | | | |
|---|---|---|---|---|---|---|---|---|---|---|---|---|---|---|---|---|---|---|---|---|---|---|---|---|---|
| Endocrinologists | .. | .. | .. | .. | 1 | .. | 1 | .. | .. | .. | .. | .. | .. | .. | .. | 1 | .. | .. | .. | .. | .. | .. | .. | .. | .. |
| Internal medicine physicians | .. | .. | .. | .. | .. | .. | .. | .. | .. | .. | .. | .. | .. | .. | .. | 2 | .. | .. | .. | .. | .. | .. | .. | .. | .. |
| Medical family doctors | .. | .. | .. | .. | .. | .. | 1 | .. | .. | .. | .. | .. | .. | .. | .. | .. | .. | .. | .. | .. | .. | .. | .. | .. | .. |
| Nephrologists | .. | .. | .. | .. | .. | .. | 1 | .. | .. | .. | .. | .. | .. | .. | .. | .. | .. | .. | .. | .. | .. | .. | .. | .. | .. |
| Pharmacists/pharmacy technicians | .. | .. | 5 | 1 | .. | .. | .. | .. | .. | .. | .. | .. | .. | .. | .. | .. | .. | .. | .. | .. | .. | .. | .. | .. | .. |
| Neurologists | .. | .. | .. | .. | .. | .. | .. | .. | .. | .. | .. | .. | .. | .. | .. | .. | .. | .. | .. | .. | .. | .. | .. | .. | .. |

*The number and type of required health personnel are determined by the level of care and specific regulations in each country.

± The corresponding letter for each HC is as indicated in Table 2.

†For Health Centre W(Guatemala), the number includes the total number of general practitioners and specialist physicians.

## Table S2. Prevalence Proportions of NCDs and Mental Health Conditions (per 1000 people)

| Country | Region | Year | Diabetes | Overweight and obesity | HBP **(2)** | Asthma | COPD **(3)** | Depression | Anxiety | Bipolar disorder | Schizophrenia | Alcohol-related diseases |
|---|---|---|---|---|---|---|---|---|---|---|---|---|
| Bolivia | City of Santa Cruz* | 2022 | 14·7 | 5·8 | 21·0 | 2·4 | 0·4 | 0·5 | 1·9 | .. | 0·2 | 0·1 |
| | City of San José de Chiquitos* | 2022 | 10·1 | 11·1 | 20·4 | 0·5 | 0·1 | 0·0 | 0·0 | .. | 0·0 | 0·0 |
| | Department of Santa Cruz* | 2022 | 14·5 | 4·1 | 23·3 | 2·0 | 0·3 | 0·3 | 1·3 | .. | 0·1 | 0·1 |
| | National± | 2022 | 37·0 | 633·0 | 159·0 | 1·2 | 0·2 | 0·4 | 1·2 | .. | 0·2 | 0·2 |
| Colombia ** | Amazonas | 2022 | 11·3 | 6·8 | 30·3 | 1·7 | 0·5 | 1·8 | 3·4 | 0·4 | 0·9 | 1·4 |
| | Bogotá D.C | 2022 | 31·4 | 24·0 | 96·6 | 6·1 | 1·3 | 8·1 | 16·4 | 2·3 | 2·9 | 0·7 |
| | Cauca | 2022 | 22·2 | 10·9 | 71·7 | 2·3 | 0·5 | 2·2 | 7·1 | 0·7 | 1·9 | 0·6 |
| | Guaviare | 2022 | 24·8 | 14·8 | 69·4 | 2·9 | 0·5 | 2·8 | 4·7 | 1·0 | 1·2 | 0·9 |
| | National | 2022 | 26·6 | 15·2 | 90·6 | 3·6 | 0·9 | 5·1 | 12·2 | 2·3 | 2·2 | 0·6 |
| Guatemala*** | Alta Verapaz | 2022 | 6·6 | 0·6 | 10·0 | 1·2 | 0·1 | 0·2 | 1·0 | 0·0 | 0·0 | 0·0 |
| | Central Guatemala | 2022 | 7·3 | 1·6 | 10·3 | 0·5 | 0·1 | 1·2 | 2·4 | 0·8 | 0·4 | 0·2 |
| | Quetzaltenango | 2022 | 9·0 | 2·1 | 10·2 | 0·6 | 0·8 | 0·5 | 3·7 | 0·0 | 0·0 | 0·9 |
| | National | 2022 | 13·9 | 1·4 | 16·8 | 1·2 | 0·2 | 0·7 | 2·6 | 0·1 | 0·1 | 0·7 |

** Data source: The prevalence calculations were based on data from the National Epidemiological Surveillance System of Bolivia (SNIS-VE).*

*** Data source: The prevalence calculations were based on data from the Health Services Information System (RIPS) and the National Administrative Department of Statistics of Colombia (DANE)*

**** Data source: The prevalence calculations were based on data from the Information Technologies Direction· Ministry of Public Health and Social Assistance of the Republic of*

*Guatemala (MSPAS). ICD10 codes: diabetes (E10-E14), obesity (E66), HPB (I10), asthma (J45), COPD (J44), depression (F32, F33), anxiety (F40, F41), bipolar disorder (F31), schizophrenia (F20), alcohol-related diseases (F10).*

*.. Missing data were either not reported or not found in the reviewed data sources.*

*± Data on the prevalence of diabetes, overweight and obesity, and high blood pressure are available up to 2021. The National Survey on Non-communicable Diseases (ENENT) was the source of this data.*

*(2) HPB: High Blood Pressure*

*(3)COPD: Chronic Obstructive Pulmonary Disease*

**Table S3. Health Centres prevalence proportions for NCDs and Mental Health Conditions (per 1000 people)**

| | HC (1) | Year | Diabetes | Overweight and | HBP (3) | Asthma | COPD (4) | Depression | Anxiety | Bipolar disorder | Schizophrenia | Alcohol-related diseases |
|---|---|---|---|---|---|---|---|---|---|---|---|---|
| Country | | | | Obesity | | | | | | | | |
| Bolivia * | A | 2022 | 103·5 | 4·1 | 76·7 | 0·2 | 0·2 | 0·2 | 0·0 | .. | .. | 0·2 |
| | B (2) | 2022 | 38·0 | 1·1 | 47·6 | 3·0 | 0·0 | .. | 0·7 | .. | .. | .. |
| | C | 2022 | .. | .. | .. | .. | .. | .. | .. | .. | .. | .. |
| | D | 2022 | .. | .. | .. | .. | .. | 136·4 | 168·3 | 63·8 | 155·0 | 13·7 |
| | E | 2022 | 69·8 | 3·2 | 104·3 | 5·1 | 5·2 | 2·4 | 2·0 | 0·6 | 2·1 | 0·2 |
| | F | 2022 | 169·8 | .. | 267·3 | 8·4 | .. | .. | .. | .. | .. | .. |
| Colombia ** | G | 2023 | 36·7 | 21·9 | 107·5 | 2·5 | 0·8 | 0·4 | 10·1 | 0·4 | 1·3 | 1·7 |
| | H | 2023 | 59·7 | | 239·4 | 8·3 | 4·1 | 21·5 | 1·7 | .. | .. | 1·7 |
| | I | 2023 | .. | .. | .. | .. | .. | 18·9 | 0·0 | 18·9 | 18·9 | 18·9 |
| | J | 2023 | 48·1 | 57·5 | 3·4 | 19·1 | 7·8 | 0·8 | 0·2 | 0·0 | 0·0 | 3·8 |

| | | | | | | | | | | | | |
|---|---|---|---|---|---|---|---|---|---|---|---|---|
| | K | 2023 | 49·3 | 62·3 | 233·2 | 7·8 | 3·2 | 5·5 | 40·0 | 2·8 | 4·8 | 0·7 |
| | L | 2023 | 9·8 | 6·8 | 40·0 | 2·3 | 0·8 | 154·8 | 183·5 | 300·6 | 432·8 | 9·8 |
| | M | 2023 | 41·6 | 11·3 | 178·9 | 4·4 | 2·2 | 23·2 | 32·8 | 1·7 | 7·1 | 0·7 |
| | N | 2023 | 30·8 | 21·6 | 70·1 | 3·6 | 1·1 | 0·5 | 7·3 | 0·1 | 1·1 | 0·5 |
| | O | 2023 | 54·0 | 22·8 | 81·4 | 3·0 | 0·8 | 2·6 | 7·2 | 0·4 | 0·3 | 1·2 |
| | P | 2022 | 33·1 | 22·2 | 82·2 | 3·6 | 0·3 | 1·9 | 3·0 | 0·5 | 1·2 | 1·5 |
| Guatemala *** | Q | 2022 | 4·2 | .. | 9·7 | .. | .. | .. | .. | .. | .. | .. |
| | R | 2022 | 1·3 | 0·3 | 4·2 | 0·6 | 0·0 | 0·0 | 0·1 | .. | 0·0 | 0·0 |
| | S | 2022 | 1·4 | .. | 2·7 | .. | .. | .. | .. | .. | .. | .. |
| | T | 2022 | .. | .. | .. | .. | .. | .. | .. | .. | .. | .. |
| | U | 2022 | 3·7 | 0·1 | 0·2 | 0·0 | 0·0 | 0·6 | 0·3 | .. | .. | 0·0 |
| | V | 2022 | 1·0 | 0·8 | 2·3 | .. | .. | 2·5 | 2·5 | 0·6 | 0·1 | 0·1 |

** Data source: The prevalence calculations were based on the record of consultations by diagnosis among the total consultations obtained at the same health centre or through each centre's network.*

*** Data source: The prevalence calculations were based on data from the health centres.*

**** Data source: The prevalence calculations of diabetes, HBP and alcohol-related diseases were based on data of vital statistics from Alta Verapaz and Quetzaltenango areas · Ministry of Public Health and Social Assistance (MSPAS). The rest of disease cases were based on data from the health centres through surveys.*

*.. Missing data were either not reported or not found in the reviewed data sources.*

*(1)HC: Health Centre*

*(2)The availability of information on the prevalence of anxiety is up to the year 2021*

*(3)HPB: High Blood Pressure*

*(4)COPD: Chronic Obstructive Pulmonary Disease*
