# Supplementary material for: Understanding the community management of long-term physical and mental health conditions in Bolivia, Colombia and Guatemala: a situational analysis
Source: BMJ Glob Health. 2026 Mar 9;11(3):e020466. doi: 10.1136/bmjgh-2025-020466 (PMC12983732; doi:10.1136/bmjgh-2025-020466)
Supplement: online supplemental file 3 [file bmjgh-11-3-s003.pdf]

BMJ Global Health Author Reflexivity Statement

Adapted from Morton, B., Vercueil, A., Masekela, R., Heinz, E., Reimer, L., Saleh, S., Kalinga, C., Seekles, M., Biccard, B., Chakaya, J., Abimbola, S., Obasi, A. and Oriyo, N. (2022), Consensus statement on measures to promote equitable authorship in the publication of research from international partnerships. Anaesthesia, 77: 264-276. https://doi.org/10.1111/anae.15597

**Study conceptualisation**	
1. How does this study address local research and policy priorities?	This situation analysis seeks to better understand and contextualise community-based care at specific territories or communities in Bolivia, Colombia and Guatemala. The results will be used to enhance research efforts for improving the community management of long-term conditions.
2. How were local researchers involved in study design?	Fully involved, researchers in Bolivia, Colombia and Guatemala co-authored the research protocol.
**Research management**	
3. How has funding been used to support the local research team(s)?	Funding for this study came from a grant that aims to support research, capacity strengthening and community engagement in Bolivia, Colombia and Guatemala.
**Data acquisition and analysis**	
4. How are research staff who conducted data collection acknowledged?	Most of the data collection was conducted by early-career researchers, who also contributed to data analysis and manuscript preparation, making their contributions substantial enough to warrant authorship.
5. How have members of the research partnership been provided with access to study data?	All the analyses were performed together with members from all teams (UK, Bolivia, Colombia and Guatemala). Anonymised data was available to all research teams.
6. How were data used to develop analytical skills within the partnership?	During the analysis process, members from all teams (UK, Bolivia, Colombia and Guatemala) took an active part in learning and sharing data analysis concepts according to each researcher's experience and knowledge.
**Data interpretation**	
7. How have research partners collaborated in interpreting study data?	Recurrent weekly meetings with researchers from the UK, Bolivia, Colombia and Guatemala were held to guide the data analysis process in each

	country and to discuss results.
**Drafting and revising for intellectual content**	
8. How were research partners supported to develop writing skills?	The early-career researcher authors drafted the manuscript with the guidance of senior researchers.
9. How will research products be shared to address local needs?	Different strategies for transferring results to the community have been taken. Strategies varied by country and region—for example, cultural activities or delivering a calendar with digestible summaries of the research findings. Also, the study results are currently being used to guide the adaptation and implementation of community-based interventions.
**Authorship**	
10. How is the leadership, contribution and ownership of this work by LMIC researchers recognised within the authorship?	First authors and almost all the co-authors have LMIC affiliations.
11. How have early career researchers across the partnership been included within the authorship team?	At least half of the authors are ECR.
12. How has gender balance been addressed within the authorship?	Authorship was granted according to pre-defined criteria and following the recommendations from the International Committee of Medical Journal Editors.
**Training**	
13. How has the project contributed to the training of LMIC researchers?	The study itself and the funding grant promoted continuous training to all members. (Weekly training sessions plus the hands-on experience)
**Infrastructure**	
14. How has the project contributed to improvements in local infrastructure?	The project has enabled the acquisition of renewed technological equipment and access to research-supporting software.
**Governance**	
15. What safeguarding procedures were used to protect local study participants and researchers?	The study protocol was submitted to review boards in the four countries. All ethics committees supervised the study's conduct. All participants went through the informed consent process. Also, before introducing the studies into vulnerable communities, they were shared and discussed with local authorities.
